# Effect of occlusal coverage depths on the precision of 3D-printed orthognathic surgical splints

**DOI:** 10.1186/s12903-022-02247-6

**Published:** 2022-06-02

**Authors:** Yipeng Wang, Peiqi Wang, Xiang Xiang, Hui Xu, Yuting Tang, Yumeng Zhou, Ding Bai, Chaoran Xue

**Affiliations:** 1 State Key Laboratory of Oral Diseases and National Clinical Research Center for Oral Diseases and Department of Orthodontics, West China Hospital of Stomatology, Sichuan University, No. 14, 3rd Section of Renmin Nan Road, Chengdu, 610041 China; 2 School of Artificial Intelligence and Automation, Huazhong University of Science and Technology, Wuhan, 430074 China

**Keywords:** Orthodontics, Orthognathic surgery, Splint, CAD/CAM

## Abstract

**Background:**

Precise orthognathic surgical splints are important in surgical-orthodontic treatment. This study aimed to propose a standardized protocol for three-dimensional (3D)-printed splints and assess the precision of splints with different occlusal coverage on the dentition (occlusal coverage depth, OCD), thus optimizing the design of 3D-printed splints to minimize the seemingly unavoidable systematic errors.

**Methods:**

Resin models in optimal occlusion from 19 patients were selected and scanned. Intermediate splints (ISs) and final splints (FSs) with 2-mm, 3-mm, 4-mm, and 5-mm OCDs were fabricated and grouped as IS-2, IS-3, IS-4, IS-5, FS-2, FS-3, FS-4, and FS-5, respectively. The dentitions were occluded with each splint and scanned as a whole to compare with the original occlusion. Translational and rotational deviations of the lower dentition and translational deviations of the landmarks were measured.

**Results:**

For vertical translation, the lower dentitions translated inferiorly to the upper dentition in most of the splints, and the translation increased as OCD got larger. Vertical translations of the dentitions in 89.47% of IS-2, 68.42% of IS-3, 42.11% of IS-4, 10.53% of IS-5, 94.74% of FS-2, 63.16% of FS-3, 26.32% of FS-4, and 21.05% of FS-5 splints were below 1 mm, respectively. For pitch rotation, the lower dentitions rotated inferiorly and posteriorly in most groups, and the rotation increased as OCD got larger. Pitch rotations of the dentitions in 100% of IS-2, 89.47% of IS-3, 57.89% of IS-4, 52.63% of IS-5, 100.00% of FS-2, 78.95% of FS-3, 52.63% of FS-4, and 47.37% of FS-5 splints were below 2°, respectively. On the other hand, the transversal and sagittal translations, roll and yaw rotations of most groups were clinically acceptable (translation < 1 mm and rotation < 2°). The deviations of ISs and FSs showed no statistical significance at all levels of coverage (*P* > 0.05).

**Conclusions:**

A protocol was proposed to generate 3D-printed ISs and FSs with normalized basal planes and standardized OCDs. Deviations of the ISs and FSs were more evident in the vertical dimension and pitch rotation and had a tendency to increase as the OCD got larger. ISs and FSs with both 2-mm and 3-mm OCD are recommendable regarding the precision relative to clinical acceptability. However, considering the fabrication, structural stability, and clinical application, ISs and FSs with 3-mm OCD are recommended for accurate fitting.

**Supplementary Information:**

The online version contains supplementary material available at 10.1186/s12903-022-02247-6.

## Background

Dentofacial deformities often manifested as disturbance of occlusal relationship and abnormal facial morphology, and generally require interdisciplinary treatment involving orthodontic and orthognathic treatment [1]. The conventional orthodontic-surgical treatment consists of three stages including presurgical orthodontics, orthognathic surgery, and postsurgical orthodontics [2–5]. Accurate diagnosis and proper treatment planning are fundamental to achieving satisfying results after orthognathic surgery. Since orthognathic surgical splints are used for transferring the preoperative surgical planning, and repositioning the dental arches and the mobile bone blocks during operation [4], the precision of the splints is important to not only the orthognathic surgery but also the postoperative stability and the ultimate outcome of the combined orthodontics and orthognathic surgery treatment [2, 6].

**Fig. 1:**
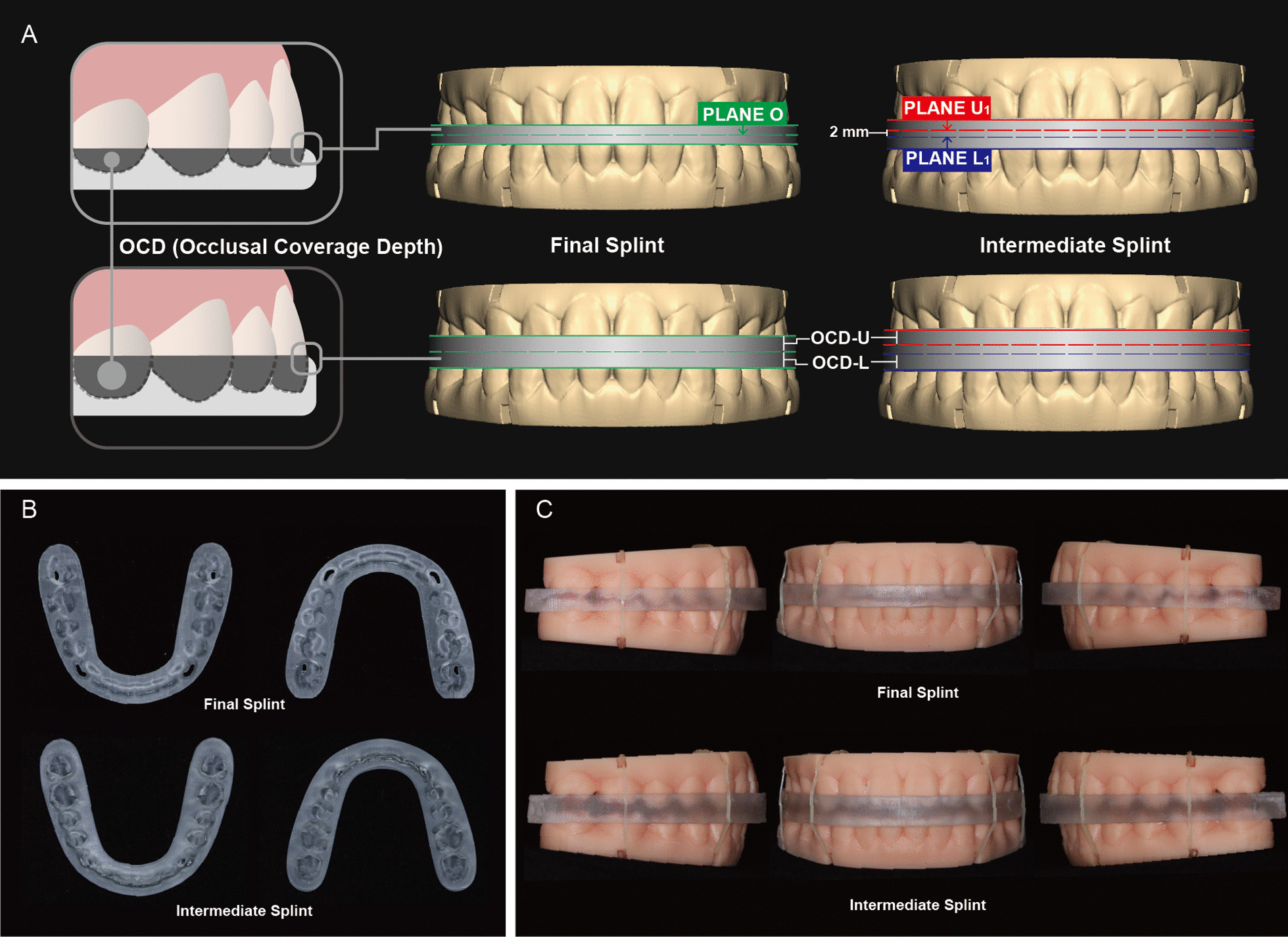
**A** Illustration of different levels of occlusal coverage depth (OCD). A lower level of OCD leads to less splint extension into the tooth embrasure (left, upper), while the higher level of OCD leads to more splint extension into the tooth embrasure (left, lower). For final splints (FSs), the upper and lower outer surfaces were determined by translated PLANE O (green dashed line). For intermediate splints (ISs), the upper and lower outer surfaces were determined by translated PLANE U_1_ (red dashed line) and PLANE L_1_ (blue dashed line), respectively. A constant 2 mm distance exists between PLANE U_1_ and PLANE L_1_. OCDs of the splints were determined by the sum of OCDs of the upper dentition (OCD-Us) and OCDs of the lower dentition (OCD-Ls). **B** Upper surface (left) and lower surface (right) of the physical splints. **C** The dentitions were occluded with the FSs (upper) or ISs (lower) and fixed with latex elastics

With the development of three-dimensional (3D) computerized technologies, virtual design and 3D printing of surgical splints have gradually become widely applied [3, 7–12]. 3D surgical splints have advantages over traditional surgical splints manufactured based on model surgery which are time-consuming and have a higher risk of reduced accuracy and reproducibility due to the tedious procedures [3, 5]. However, the potential for systematic errors exists during the process of digital scanning of the dentition [13–15] or 3D printing of the surgical splint [11, 16–19], and thus the printed surgical splint may not always perfectly match the dentitions, leading to deviations in jaw positioning [20–22]. Moreover, since the surgical splint inevitably extends to the embrasure undercuts, it may suffer resistance during intraoperative placement, and the imperfect seating of the surgical splint on the dentition can also lead to differences in jaw position from virtual planning [23, 24]. The occlusal coverage depth (OCD), i.e., the depth from the splint outer surface to the occlusal contact, determines the extension of the splints into the embrasure undercuts that hinder surgical splint seating and therefore influences the fitting accuracy of the splints (Fig. 1A).

Therefore, this study aimed to assess the precision of surgical splints with different levels of OCD and improve the design of 3D-printed surgical splints accordingly, to compensate for the seemingly unavoidable errors. The hypothesis was that OCD would affect the precision of both intermediate splints (ISs) and final splints (FSs).

## Methods

### Inclusion of dental models

The resin models with grooves for intermaxillary bonding (Fig. 1C) from 19 patients were scanned with a desktop 3D scanner (R900; 3Shape, Copenhagen, Denmark). The inclusion criteria were: a complete permanent dentition with normal crown morphology, an optimal occlusal relationship, a curve of spee of less than 2 mm, and crowding and spacing of less than 2 mm in the upper and lower dentitions [25]. The project was under the approval of the local institutional ethical committee (WCHSIRB-CT-2021-310).

### Design and fabrication of the surgical splints

Three standard planes were generated to form the splints in Geomagic Studio 2013 software (Geomagic, Morrisville, NC, USA). To be specific, functioning areas, i.e., lingual surfaces of the maxillary anterior teeth, labial-incisal edges of the mandibular anterior teeth, and functional cusps of the posterior dentition, were manually selected using the Select Visible Only mode. With the selected areas in the upper and lower dentition, two planes (PLANE U_0_ and PLANE L_0_) were created respectively, using the Best Fit Alignment approach. In this way, PLANE U_0_ and PLANE L_0_ represented the average occlusal contact areas of the upper and lower dentitions, respectively, and were not necessarily parallel. PLANE O was generated from PLANE U_0_ and PLANE L_0_ using the 2-Plane Average approach when the two dentitions occluded in their designed positions, representing the average plane of the PLANE U_0_ and PLANE L_0_. The digital models and the three planes were imported into Geomagic Freeform software (Geomagic, Morrisville, NC, USA). U-shaped blank splints were generated with the contours designed on the models.

For the FSs, the upper and lower dentitions were in the original occlusion, and the upper and lower outer surfaces of FSs were generated by translations of PLANE O. Upper OCD (OCD-U) of the FS was defined as the vertical height between the upper outer surface of the FS and PLANE O, while lower OCD (OCD-L) of the FS was the vertical height between and the lower outer surface of the FS and PLANE O (Fig. 1A). Because PLANE O was the average plane of PLANE U_0_ and PLANE L_0_ that represented the average occlusal contact areas of the upper and lower dentitions, OCD-U and OCD-L defined by distances between PLANE O and the translated PLANE O represented the average coverage depth of the FS onto the dentition.

Meanwhile, for the ISs, the upper and lower dentitions together with PLANE U_0_ and PLANE L_0_ were translated perpendicular to PLANE O, up and down by 1 mm respectively, generating PLANE U_1_ and PLANE L_1_. The 2 mm distance was to increase the thickness of the ISs and separate the upper and lower dentitions as in multiple clinical scenarios. Afterward, the upper and lower surfaces of ISs were generated by translations of PLANE U_1_ and PLANE L_1_, respectively. OCD-U of the IS was defined as the vertical height between the upper outer surface of the IS and PLANE U_1_, while OCD-L of the IS was the vertical height between the PLANE L_0_ and the lower outer surface of the IS, representing the average coverage depth of the IS onto the dentition. Because PLANE U_1_ and PLANE L_1_ were generated by translating PLANE U_0_ and PLANE L_0_ respectively, PLANE U_1_ and PLANE L_1_ were not necessarily parallel just like PLANE U_0_ and PLANE L_0_, and there was a constant vertical distance of approximately 2 mm between PLANE U_1_ and PLANE L_1_ for all ISs (Fig. 1A).

Indentations of the dentitions on the splints were created by applying a Boolean operation to the dentitions in their designed positions. The ISs and FSs were divided into four groups, respectively, with different OCDs, OCD-Us, OCD-Ls, and thicknesses as shown in Table 1.

**Table 1:** Features of groups divided by different occlusal coverage depth (OCD)

	Intermediate Splint				Final Splint			
Group	IS-2	IS-3	IS-4	IS-5	FS-2	FS-3	FS-4	FS-5
OCD-U (mm)	1	1.5	2	2.5	1	1.5	2	2.5
OCD-L (mm)	1	1.5	2	2.5	1	1.5	2	2.5
OCD (mm)	2	3	4	5	2	3	4	5
Thickness (mm)	4	5	6	7	2	3	4	5

*Upper OCD (OCD-U)* was defined by the distance between the upper outer surface of the splint and PLANE U_1_ for the intermediate splint (IS), or PLANE O for the final splint (FS). *Lower OCD (OCD-L)* was defined by the distance between the lower outer surface of the splint and PLANE L_1_ for the intermediate splint, or PLANE O for the final splint. *OCD* was determined by the sum of OCD-U and OCD-L, for either IS or FS, representing the total average coverage depth of the splints onto the dentition. The thickness of the splint was determined by the distance between the upper and lower outer surfaces of the splints. The thickness of the IS was approximated because the upper and lower outer surfaces of the IS were not always parallel since they were generated from translated PLAN U_1_ and PLANE L_1_

Finally, the splints were 3D-printed (NOVA3D Bene3; Nova Intelligent Technology Co., Ltd., Shenzhen, China) with an accuracy of 0.01 mm and a build-up layer thickness of 0.05 mm (Fig. 1B).

### Precision evaluation of the surgical splints

The upper and lower dentitions of the resin models were occluded on the 3D-printed ISs and FSs, respectively. Then they were fixed with latex elastics (3/16 in, 4.5 oz; American Orthodontics, American Orthodontics, Sheboygan, Wisconsin, USA) between the upper and lower grooves (Fig. 1C).

**Fig. 2:**
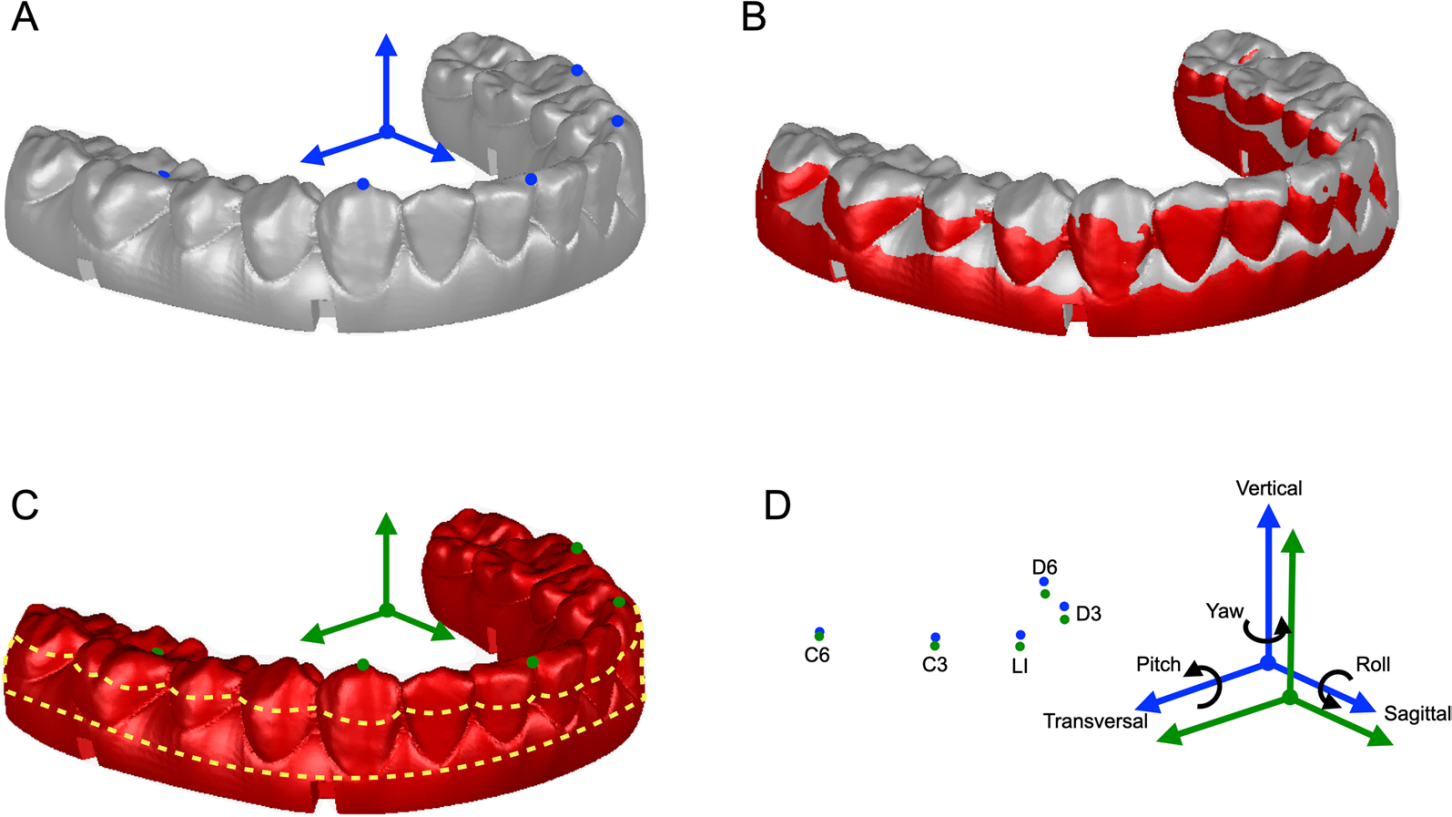
Semiautomatic measurement of the deviation in six degrees of freedom. **A** Lower dentition in the original position (gray), with the landmarks selected (blue points). The coordinate system was constructed by taking PLANE O as the horizontal plane, X-axis determined by the projection of the connecting line of the contact points of left/right upper first and second premolars onto the horizontal plane, the origin as the midpoint of the projected line, the mid-sagittal plane defined as passing through the origin and perpendicular to the X-axis, and the coronal plane passing through the origin and perpendicular to both the horizontal plane and the mid-sagittal plane. **B** By selecting the same region on the upper dentition (not shown in the illustration), the scanned model set with the scanned occlusion was registered to the position of the original dentition. **C** By selecting the same region on the lower dentition (the actual scanned area that was not covered by the splint, circled by the yellow dashed line), the original lower dentition with the coordinate system and landmarks was registered to the scanned lower dentition (red), generating a new coordinate system (green) and five landmarks (green points) with the position of the scanned dentition. **D** The deviations in translation (transversal, sagittal, and vertical deviations) between the coordinate systems and landmarks (LI, C3, D3, C6, D6) in the scanned (green) and original (blue) positions, and deviations in rotation (pitch, roll, yaw) between the coordinate systems in the scanned (green) and original (blue) positions, were automatically computed. The directions of the arrows represented the positive directions of deviations

Each set of the fixed models was scanned using the desktop 3D scanner (Shining 3D AutoScan DS100; Shining 3D, Hangzhou, China). In Geomagic Studio 2013 software, a coordinate system was constructed and five landmarks (LI, C3, D3, C6, D6) were selected on each original model set (Fig. 2A). Specifically, LI represented the most mesial point of the tip of the crown of each lower central incisor; C3 and D3 represented the most superior points of the right and left lower canines, respectively; and C6 and D6 represented the most inferior points of the central fossae of the right and left first lower molars, respectively. The original model with the coordinate system was registered to the position of the scanned model by selecting the same regions on the scanned and original dentitions by means of Global Registration (Fig. 2B and C). In this way, the deviation of the occlusion could be represented by the difference between the position of the original and the scanned lower dentitions. Six parameters including the transversal, sagittal, vertical translations, and the pitch, roll, and yaw rotations of the lower dentition as represented by the positional differences of two coordinate systems, as well as the translational deviations of five landmarks (LI, C3, D3, C6, D6), were automatically computed (Fig. 2D) [26–29]. All measurements were performed twice by the same investigator with a 2-week interval to evaluate the reproducibility of measurements. The average value of the repeated measurements was used for further statistical analysis.

### Statistical analysis

Bland–Altman plots were used to assess the intra-observer reproducibility, established with Medcalc software (version 11.4.2.0; Medcalc Software, Mariakerke, Belgium) [30]. Statistical analyses were performed in SPSS software. Absolute translations and rotations between the scanned and original lower dentitions, and absolute translations of the landmarks were presented. Shapiro–Wilk test was applied to assess normality of data distribution, and differences between groups were analyzed using one-way analysis of variance (ANOVA) followed by Tukey post hoc analysis (for normally distributed data) or Kruskal–Wallis H with Nemenyi test (for nonnormally distributed data). A level of *P* = 0.05 was set for significance. With a total sample size of 76 (19 splints per group), the power analysis using Wilcoxon matched-pairs signed-rank test (2-tailed) indicated 82.34% power in detecting small effect sizes at a significance level of 0.05 (Cohen's d = 0.4) (G*Power 3.1.9.3).

## Results

### Intra-observer reproducibility of the measurement.

**Fig. 3:**
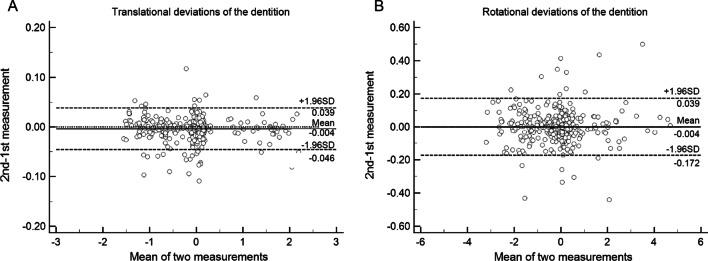
Bland–Altman plots showing the intra-reproducibility of the measurements of **A** translational deviations and **B** rotational deviations

The Bland–Altman plots suggested mean differences of − 0.004 mm and − 0.004° and 95% limits of agreement ranging from − 0.046 mm to 0.039 mm and − 0.172° to 0.039° between repeated measurements of translational and rotational deviations (Fig. 3), respectively, indicating high reproducibility.

### Vertical translation of the lower dentitions to the upper dentitions.

**Fig. 4:**
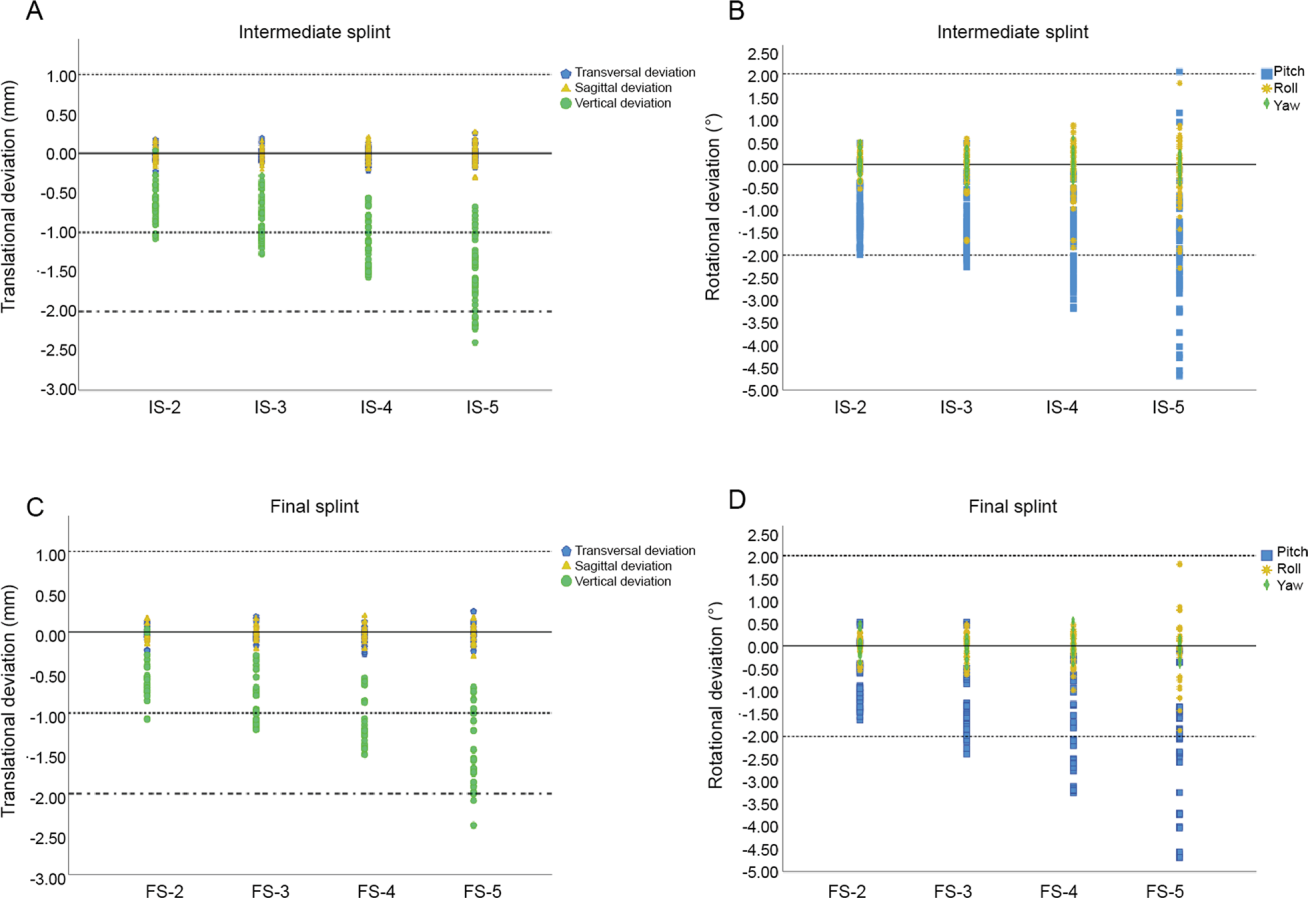
Scatter diagrams of **A** translational deviations of the intermediate splints, **B** rotational deviations of the intermediate splints, **C** translational deviations of the final splints, **D** rotational deviations of the final splints

**Fig. 5:**
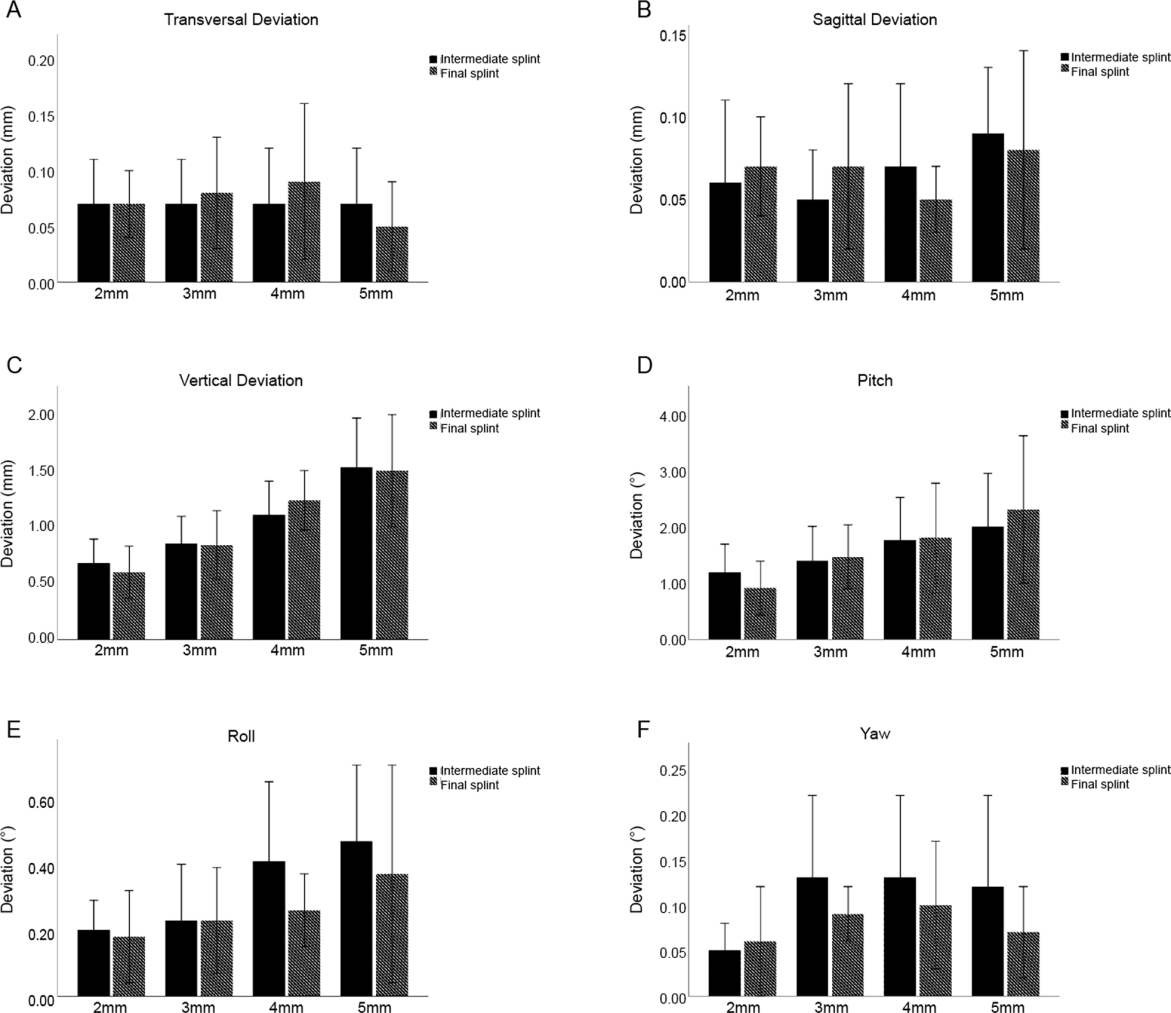
Comparison of absolute deviations between intermediate and final splints with different levels of OCDs **A** in transversal dimension; **B** in sagittal dimension; **C** in vertical dimension; **D** in pitch rotation; **E** in roll rotation; **F** in yaw rotation. *P* values of the comparison are separately listed in Additional file 1: Table S1

Translational deviations mainly existed in the vertical dimension and the values rose as the OCD increased (Fig. 4). There were statistically significant differences between all groups of ISs (*P* < 0.05), with means ± standard deviations (SDs) of 0.67 ± 0.21, 0.84 ± 0.24, 1.09 ± 0.30, and 1.51 ± 0.43 mm for IS-2, IS-3, IS-4, and IS-5, respectively (Fig. 5, Table 2 and S1). Meanwhile, among the FSs, significant differences were found between all groups (*P* < 0.05) except between FS-2 and FS-3, and the means ± SDs for FS-2, FS-3, FS-4, and FS-5 were 0.59 ± 0.23 and 0.83 ± 0.30, 1.22 ± 0.26, 1.48 ± 0.49 mm, respectively (Fig. 5, Table 2 and Additional file 1: Table S1). Consistently, evident deviations of the landmarks (L1, C3, D3, C6, D6) were found in the vertical dimension, with mean values ranging from 0.50 to 2.27 mm (Table 2).

**Table 2:** Deviations of the mandibular dentition and the landmarks between the scanned dentition and the original dentition

Measurement	Intermediate Splint				Final Splint			
	2 mm	3 mm	4 mm	5 mm	2 mm	3 mm	4 mm	5 mm
	Mean ± SD or Median ± Quartile^#^				Mean ± SD or Median ± Quartile^#^			
**Dentition**								
Translational deviation (mm)								
X	0.07 ± 0.04	0.07 ± 0.04	0.07 ± 0.05	0.07 ± 0.05	0.07 ± 0.03^#^	0.08 ± 0.05	0.09 ± 0.07	0.05 ± 0.04^#^
Y	0.06 ± 0.05	0.05 ± 0.03^#^	0.07 ± 0.05	0.09 ± 0.04^#^	0.07 ± 0.03^#^	0.07 ± 0.05	0.05 ± 0.02^#^	0.08 ± 0.06^#^
Z	0.67 ± 0.21	0.84 ± 0.24	1.09 ± 0.30	1.51 ± 0.43	0.59 ± 0.23	0.83 ± 0.30	1.22 ± 0.26^#^	1.48 ± 0.49
Rotational deviation (°)								
Pitch	1.18 ± 0.49	1.38 ± 0.60	1.75 ± 0.74	1.98 ± 0.93	0.90 ± 0.47	1.45 ± 0.56	1.78 ± 0.96	2.28 ± 1.29
Roll	0.20 ± 0.09^#^	0.23 ± 0.17^#^	0.41 ± 0.24^#^	0.47 ± 0.23^#^	0.18 ± 0.14	0.23 ± 0.16	0.26 ± 0.11^#^	0.37 ± 0.33^#^
Yaw	0.05 ± 0.03^#^	0.13 ± 0.09	0.13 ± 0.09	0.12 ± 0.10	0.06 ± 0.06^#^	0.09 ± 0.03^#^	0.10 ± 0.07^#^	0.07 ± 0.05^#^
**Landmarks**								
Translational deviation (mm)								
LI-X	0.09 ± 0.05	0.06 ± 0.04^#^	0.04 ± 0.04^#^	0.07 ± 0.06^#^	0.01 ± 0.01^#^	0.10 ± 0.06^#^	0.08 ± 0.03^#^	0.06 ± 0.03^#^
LI-Y	0.07 ± 0.05	0.04 ± 0.03^#^	0.08 ± 0.05	0.09 ± 0.04^#^	0.07 ± 0.02	0.07 ± 0.04^#^	0.06 ± 0.02^#^	0.09 ± 0.07
LI-Z	1.08 ± 0.30	1.20 ± 0.29	1.70 ± 0.41	2.08 ± 0.62	0.88 ± 0.32	1.31 ± 0.45	1.75 ± 0.49	2.27 ± 0.78
C3-X	0.08 ± 0.05	0.08 ± 0.06	0.06 ± 0.04^#^	0.08 ± 0.07	0.07 ± 0.05	0.09 ± 0.06^#^	0.07 ± 0.04^#^	0.06 ± 0.04^#^
C3-Y	0.08 ± 0.05	0.05 ± 0.04^#^	0.09 ± 0.06	0.12 ± 0.04^#^	0.09 ± 0.06^#^	0.06 ± 0.06^#^	0.04 ± 0.04	0.11 ± 0.08^#^
C3-Z	0.96 ± 0.27	1.22 ± 0.35	1.58 ± 0.40	2.02 ± 0.63	0.79 ± 0.30	1.19 ± 0.44	1.59 ± 0.43	2.07 ± 0.72
D3-X	0.09 ± 0.05	0.08 ± 0.06	0.06 ± 0.04^#^	0.08 ± 0.06	0.10 ± 0.09^#^	0.09 ± 0.06^#^	0.07 ± 0.03^#^	0.06 ± 0.04^#^
D3-Y	0.07 ± 0.04	0.06 ± 0.03^#^	0.07 ± 0.03	0.08 ± 0.05^#^	0.07 ± 0.03	0.07 ± 0.05^#^	0.05 ± 0.03	0.08 ± 0.04
D3-Z	1.00 ± 0.28	1.19 ± 0.27	1.50 ± 0.22	1.85 ± 0.56	0.82 ± 0.28	1.61 ± 0.42	1.48 ± 0.35	1.19 ± 0.52
C6-X	0.07 ± 0.04^#^	0.08 ± 0.05^#^	0.08 ± 0.05^#^	0.09 ± 0.06	0.06 ± 0.02^#^	0.06 ± 0.03^#^	0.08 ± 0.06	0.07 ± 0.05
C6-Y	0.06 ± 0.06^#^	0.07 ± 0.07	0.10 ± 0.06	0.16 ± 0.11^#^	0.11 ± 0.07	0.09 ± 0.07	0.09 ± 0.03	0.13 ± 0.09^#^
C6-Z	0.50 ± 0.26	0.69 ± 0.36	0.90 ± 0.40	1.41 ± 0.69	0.50 ± 0.22	0.68 ± 0.32	0.91 ± 0.35^#^	1.21 ± 0.55
D6-X	0.07 ± 0.04^#^	0.09 ± 0.04	0.07 ± 0.03	0.10 ± 0.08	0.06 ± 0.03^#^	0.08 ± 0.06^#^	0.07 ± 0.04	0.09 ± 0.08^#^
D6-Y	0.03 ± 0.04^#^	0.11 ± 0.07^#^	0.10 ± 0.07^#^	0.07 ± 0.05	0.09 ± 0.06	0.11 ± 0.06^#^	0.10 ± 0.05^#^	0.10 ± 0.04
D6-Z	0.55 ± 0.19	0.64 ± 0.24	0.84 ± 0.33	1.13 ± 0.43	0.51 ± 0.18	0.66 ± 0.27	1.00 ± 0.14	1.19 ± 0.52

Absolute values of deviations were used for statistical analysis. Means ± standard deviations (SDs) of the absolute values were given for normally distributed data, while medians ± quartiles were presented for the nonnormally distributed data (marked with ^#^). X, transversal translation of the landmarks and the dentition; Y, sagittal translation of the landmarks and the dentition; Z, vertical translation of the landmarks and the dentition. LI, the most mesial point of the tip of the crown of each lower central incisor. C3, the most superior point of the right lower canine. D3, the most superior point of the left lower canine. C6, the most inferior point of the central fossa of the right first lower molar. D6, the most inferior point of the central fossa of the left first lower molar

When considering the clinically acceptable range, most of the ISs and FSs with 2-mm and 3-mm OCDs, that is, 89.47% of the IS-2, 68.42% of the IS-3, 94.74% of the FS-2, and 63.16% of the FS-3 splints, led to clinically acceptable (< 1 mm) vertical deviations of the lower dentitions relative to the upper dentitions (Table S2). Moreover, the two-sided 95% confidence intervals (CI) of the means of deviations in the IS-2, IS-3, FS-2, and FS-3 groups were below the 1 mm range (Table S2). Meanwhile, only less than half of IS-4, IS-5, FS-4, and FS-5 splints led to deviations of less than 1 mm (Table 3).

**Table 3:** Frequency of splints with clinically acceptable deviations^*^

Measurement	Intermediate Splint				Final Splint			
	2 mm	3 mm	4 mm	5 mm	2 mm	3 mm	4 mm	5 mm
**Dentition**								
Translational deviation (mm)								
X	100.00%	100.00%	100.00%	100.00%	100.00%	100.00%	100.00%	100.00%
Y	100.00%	100.00%	100.00%	100.00%	100.00%	100.00%	100.00%	100.00%
Z	89.47%	68.42%	42.11%	10.53%	94.74%	63.16%	26.32%	21.05%
Rotational deviation (°)								
Pitch	100.00%	89.47%	57.89%	52.63%	100.00%	78.95%	52.63%	47.37%
Roll	100.00%	100.00%	100.00%	94.74%	100.00%	100.00%	100.00%	100.00%
Yaw	100.00%	100.00%	100.00%	100.00%	100.00%	100.00%	100.00%	100.00%

*Translational deviations < 1 mm and rotational deviations < 2° were considered clinically acceptable

### Pitch rotation of the lower dentitions to the upper dentitions.

With respect to rotations, pitch rotations were higher than roll and yaw rotations and tended to increase as the OCDs raised (Fig. 4). Among the ISs, the means ± SDs of pitch deviations were 1.18 ± 0.49°, 1.38 ± 0.60°, 1.75 ± 0.74°, and 1.98 ± 0.93° for IS-2, IS-3, IS-4, and IS-5, respectively, while statistically significant difference was only found between IS-2 and IS-5 (Fig. 5, Table 2 and Additional file 1: Table S1). Meanwhile, for the FSs, deviations in the FS-2 were significantly lower than in all the other groups, with a mean ± SD of 0.90 ± 0.47° (Fig. 5, Table 2 and Additional file 1: Table S1). The relatively large deviations in the pitch rotation were in line with the tendency that vertical deviations of the anterior landmarks (L1, C3, and D3) were larger than those of the posterior landmarks (C6 and D6) (Table 2). To be specific, mean deviations of the anterior landmarks ranged from 0.79 to 2.27 mm and those of the posterior landmarks ranged from 0.50 to 1.41 mm.

When compared with the clinical acceptable range, all the IS-2 and FS-2 splints had pitch deviations below 2°, while 89.47% of IS-3 and 78.95% of FS-3 had pitch deviations below 2°. Moreover, the two-sided 95% CIs of the means of pitch in IS-2, IS-3, FS-2, and FS-3 were below the 2° range (Additional file 2: Table S2). Only a small portion of the IS-4, IS-5, FS-4, and FS-5 splints had clinically acceptable deviations (Table 3).

### Other deviations of the lower dentitions to the upper dentitions

Transversal and sagittal translations in almost all groups were clinically acceptable (< 1 mm) with the mean values ranging from 0.05 mm and 0.09 mm. Meanwhile, roll and yaw rotations in all groups were clinically acceptable (< 2°) with the mean values ranging from 0.05° to 0.47° except FS-5 with a clinical acceptability rate of 94.74% and a mean ± SD of 0.37 ± 0.33° in roll (Tables 2 and 3).

### Comparison between the ISs and FSs with the same OCD

Noteworthily, although the ISs and FSs were generated in different manners (Fig. 1), the deviations of ISs and FSs showed no statistical significance at every level of OCD (Fig. 5, Table S1).

## Discussion

The purpose of this study was to evaluate the precision of ISs and FSs with different OCDs, further to minimize the inevitable technical errors. We proposed a protocol comprising the establishment of normalized basal planes and standardized OCDs. It was found that the deviations of both ISs and FSs mainly existed in the vertical dimension and pitch rotation, and tended to increase as the OCD got larger.

In recent years, computer-aided design and computer-aided manufacturing (CAD/CAM) splints have been widely applied in surgical-orthodontic treatment. The splints are often produced in professional software by a Boolean operation which subtracts the digital dentition from the blank splints [3, 8, 31]. Theoretically, splints generated by the Boolean operation have exact indentations of the digital models and thus can be fully fitted on the model. Nevertheless, the digital models may not coincide exactly with the physical ones, especially in the interproximal areas due to scanning errors, and the printed surgical splints can be different from the virtual design because of the printing errors [11, 13–19]. Therefore, the 3D-printed surgical splints do not always fit well. It is thus important to improve the match via optimized design.

As the undercut areas, especially embrasure undercuts, often hinder splint insertion and compromise the fit of the splint, optimized designs could be considered accordingly. To this end, Ye et al. [26] produced single-sided surgical splints from offset models to avoid insertion into the undercuts and found that splints generated from offset dental models fit better on the teeth than those from no-offset ones. In the present study, we created a standardized protocol to generate double-sided splints to study the influence of OCD on splint fit since it may affect the engagement of the undercut areas. Currently, most of the existing CAD/CAM splint fabricating protocols depend upon the automatic operations in the software and/or arbitrary or random trimming. Even though some of the software provide modifiable thicknesses or angles, the procedure of splint design remains ruleless and ambiguous [32]. Here we managed to create a set of rules to generate the splints. Functioning areas of the dentition were selected to create the basal planes. For the ISs, considering the clinical scenarios such as maxilla uplift in which the space could be large between the mandible and the designed position of the maxilla, and the upper and lower dentitions would always be separated [33], the splints were given a constant initial thickness of 2 mm (Fig. 1B, Table 1). And the upper and lower splint surfaces were determined by the translated upper and lower functional planes (PLANE U_1_ and PLANE L_1_). As for the FSs, because the upper and lower functional planes always intersect and no initial thickness was added, translated functional planes may constitute vulnerable spots or even fail to form splints. The upper and lower splint surfaces were therefore determined by translating the average plane of the two functional planes (PLANE O). This standardized procedure was technically insensitive and provided high consistency and reproducibility.

Since the clinical evaluation of the treatment comprises measurement in six degrees of freedom [34], both translational and rotational parameters should be involved to facilitate better prediction of the splint precision and the resulting treatment outcome. Multiple previous studies have compared the movement of bone blocks guided by 3D printed splints with conventional splints and reported acceptable accuracy of the 3D splints in translational and rotational orientations [22, 35]. Specifically, Shqaidefet al. [35] suggested that the deviation existed mostly in the vertical direction. As conventional splints may also result in deviations, the comparison results might have underestimated the magnitude of the errors. It is thus meaningful to evaluate the fit of the splints or splint-derived deviation in comparison with the virtual design other than with the conventional splints. To quantify the splint fit, Gateno et al. [36] used impression material to examine the space between the teeth and the splint. On this basis, Ye et al. [26] measured the airspace by weighing the overflowing impression material. They also managed to calculate the shell-to-shell deviation by measuring the 3D euclidean distances between the scanned splints and the original digital splints. In the present study, we aimed to evaluate the deviation in occlusion derived from splint fit, and included measurements in translation and rotation. To rationalize and simplify the measurements, the scanned models with splints in place were registered to the original models by selecting the maxillary dentition, and any deviation produced by the splint would be transferred to the lower dentition. In this way, the measurements comprising translation and rotation were feasible and comprehensive.

Our results suggested that deviations of the lower dentitions relative to the upper dentitions were more evident in the vertical dimension and pitch rotation, and nearly all the values of the deviations were negative. This indicated incomplete seating of the splint on the occlusal surfaces and poorer fitting in the anterior area than the posterior dentition. Meanwhile, the lower dentition seemed to deviate posteriorly according to the negative values in the sagittal dimension. In clinical practice, the posterior deviation can be further magnified because the vertical deviation of the dentition affects not only the vertical position of the mandible but also the sagittal relation between the two jaws due to the clockwise mandible rotation [37]. We assume that the directional characteristics in the sagittal dimension and pitch rotation might be partially attributed to the manner of fixation in which the force was centered in the relatively posterior section (Fig. 1C). Therefore, additional anterior fixation in clinical practice could be recommended.

According to our results, IS-2 and FS-2 (2-mm-OCD splints) gained the best fit with the lowest mean deviation. However, splints with both 2-mm and 3-mm OCDs showed their superiority in clinical application, as the majority of 2-mm-OCD and 3-mm-OCD splints led to clinically acceptable deviations below 1 mm and 2°, and the 95% CIs of means of deviations for splints with both 2-mm and 3-mm OCDs were below the threshold [38].

We consider 3-mm OCD, instead of 2-mm OCD, as the most appropriate for IS and FS fabrication after thorough consideration. First, the dentitions we included had flattened occlusal curves which are sometimes difficult to achieve. For patients with steep occlusal curves, 2-mm OCD seems to be relatively shallow and may cause instability during surgical retention. Also, 3 mm-OCD-splints are less prone to deformation and breakage.

It may be argued that the millimeter-range deviations can be disguised in clinical practice as the mandible rotates counterclockwise after removal of the splints with poor fit. However, although the deviations may be partially concealed, the post-surgical occlusion can differ from the designed one since the adjustment of occlusion is exquisite. Moreover, orthognathic surgery is highly delicate and comprises multiple steps, and errors may therefore be accumulated. Other than surgical splints, deviations may also be derived from other surgical procedures or devices [28]. To minimize the deviation of the actual post-surgical position from the designed one, it is important to improve precision in every step of the surgery. It is therefore important to exploit splint design for a better fit. The thresholds were set at 1 mm and 2°, respectively, in the present study [4, 22, 28].

It is worth noting that the standard protocol proposed in this study included standardized dentitions with optimal occlusal relationship [25], and therefore may not apply to dentitions with severe malocclusion or discrepant arch forms, especially in early surgery or surgery-first cases [39]. For instance, cases with excessively deep spee curves may not be fully covered by the splints generated from the translated average occlusal planes (standard planes). Also, tooth inclination needs to be paid special attention to because the inclined teeth may impede the splint seating due to the lack of path of insertion [40]. Further studies on cases with nonstandard dentitions are still in need. Individualized surgical splints with different designs such as different coverage depth of each tooth or different undercut offsets in different sections of the splints may be designed to better coordinate the fitting of all areas. Approaches to reduce the undercut area of the model or increase the interspace between the splint and the dentition may also be adopted.

Moreover, studies on other influencing factors of splint precision, such as fixation manner which might cause slippage of the splint, may further improve the splint design. The standardized splints could be modified to apply to special cases such as patients with edentulous areas who call for combined orthodontic-orthognathic-prosthetic treatments [41], and patients requiring specific surgical procedures that would change the occlusion during surgery, for instance, multiple-segment osteotomy [42]. Since other surgical devices may also result in deviations, this study on surgical splints may also inspire forthcoming studies on other CAD/CAM surgical aids, such as osteotomy guide, screw hole-positioning guide, and plate-positioning guide.

## Conclusion

The 3D-printed ISs and FSs led to more evident deviations in the vertical dimension and pitch rotation, and splints with 2-mm and 3-mm OCDs fit better than those with 4-mm and 5-mm OCDs. ISs and FSs with both 2-mm and 3-mm OCD are recommendable regarding the precision relative to clinical acceptability. However, considering the fabrication, structural stability, and clinical application, ISs and FSs with 3-mm OCD are recommended for accurate fitting. The standardized protocol and the results could be an inspiration for the generation of not only orthognathic surgical splints but also other CAD/CAM splint-like devices. Research to further improve the fit of splints is still needed.

## Supplementary Information

**Additional file 1**. *P*-values for paired comparison of the deviations.

**Additional file 2**. 95% confidence intervals (CIs) of the means or medians of the deviations of the mandibular dentition.

## Data Availability

All data generated or analyzed during the current study are available from the corresponding author on reasonable request.

## Abbreviations

3D: Three dimension, three dimensional
OCD: Occlusal coverage depth
FS: Final splint
OCD-U: Upper occlusal coverage depth
OCD-L: Lower occlusal coverage depth
IS: Intermediate splint
SD: Standard deviation
CAD/CAM: Computer-aided design and computer-aided manufacturing
