# Supplementary material for: Effect of occlusal coverage depths on the precision of 3D-printed orthognathic surgical splints
Source: BMC Oral Health. 2022 Jun 2;22:218. doi: 10.1186/s12903-022-02247-6 (PMC9161535; doi:10.1186/s12903-022-02247-6)
Supplement: Supplementary file 1 — Additional file 1. P-values for paired comparison of the deviations. [file 12903_2022_2247_MOESM1_ESM.docx]

**Table S1. *P*-values for paired comparison of the deviations^*^**

| ***P*-value** |  | **Intermediate Splint** | | | | **Final Splint** | | | |
| --- | --- | --- | --- | --- | --- | --- | --- | --- | --- |
| **Vertical Deviation** |  |  |  |  |  |  |  |  |  |
|  |  | IS-1 | IS-2 | IS-3 | IS-4 | FS-1 | FS-2 | FS-3 | FS-4 |
| Intermediate Splint | IS-1 | / | P<0.05 | P<0.05 | P<0.05 | 0.274 | / | / | / |
|  |  |  |  |  |  |  |  |  |  |
|  | IS-2 |  | / | P<0.05 | P<0.05 | / | 0.886 | / | / |
|  | IS-3 |  |  | / | P<0.05 | / | / | 0.668 | / |
|  | IS-4 |  |  |  | / | / | / | / | 0.862 |
| Final Splint | FS-1 |  | / | / | / | / | 0.177 | 0.000 | 0.000 |
|  | FS-2 | / |  | / | / |  | / | 0.043 | 0.000 |
|  | FS-3 | / | / |  | / |  |  | / | 0.017 |
|  | FS-4 | / | / | / |  |  |  |  | / |
| **Pitch Deviation** |  |  |  |  |  |  |  |  |  |
|  |  | IS-1 | IS-2 | IS-3 | IS-4 | FS-1 | FS-2 | FS-3 | FS-4 |
| Intermediate Splint | IS-1 | / | 0.841 | 0.144 | 0.031 | 0.078 | / | / | / |
|  | IS-2 |  | / | 0.555 | 0.210 | / | 0.707 | / | / |
|  | IS-3 |  |  | / | 0.915 | / | / | 0.790 | / |
|  | IS-4 |  |  |  | / | / | / | / | 0.288 |
| Final Splint | FS-1 |  | / | / | / | / | 0.177 | 0.000* | 0.000* |
|  | FS-2 | / |  | / | / |  | P<0.05 | P<0.05 | P<0.05 |
|  | FS-3 | / | / |  | / |  |  | P>0.05 | P>0.05 |
|  | FS-4 | / | / | / |  |  |  |  | P>0.05 |

^*^*P*-values generated from one-way ANOVA were given as specific values, while P-values from Kruskal-Wallis H with Nemenyi test were given as significant (*P* < 0.05) or statistically not significant (*P* > 0.05). Statistically significant values were underlined.
