# Supplementary material for: Effect of occlusal coverage depths on the precision of 3D-printed orthognathic surgical splints
Source: BMC Oral Health. 2022 Jun 2;22:218. doi: 10.1186/s12903-022-02247-6 (PMC9161535; doi:10.1186/s12903-022-02247-6)
Supplement: Supplementary file 2 — Additional file 2. 95% confidence intervals (CIs) of the means or medians of the deviations of the mandibular dentition. [file 12903_2022_2247_MOESM2_ESM.docx]

**Table S2. 95% confidence intervals (CIs) of the means or medians of the deviations of the mandibular dentition.**

|  | **Intermediate Splint** | | | |  | **Final Splint** | | | |
| --- | --- | --- | --- | --- | --- | --- | --- | --- | --- |
|  | **2 mm** | **3 mm** | **4 mm** | **5 mm** |  | **2 mm** | **3 mm** | **4 mm** | **5 mm** |
| **Measurement** | **95% CI** | | | |  | **95% CI** | | | |
| **Translational deviation (mm)** |  |  |  |  |  |  |  |  |  |
| X | 0.04-0.09 | 0.05-0.09 | 0.04-0.09 | 0.05-0.10 |  | 0.05-0.11 | 0.05-0.10 | 0.05-0.12 | 0.05-0.12 |
| Y | 0.04-0.09 | 0.04-0.09 | 0.05-0.10 | 0.07-0.14 |  | 0.06-0.10 | 0.04-0.09 | 0.05-0.10 | 0.06-0.13 |
| Z | 0.57-0.77 | 0.72-0.96 | 0.94-1.24 | 1.30-1.72 |  | 0.48-0.70 | 0.67-0.98 | 0.99-1.29 | 1.25-1.72 |
| **Rotational deviation (°)** |  |  |  |  |  |  |  |  |  |
| Pitch | 0.94-1.41 | 1.08-1.68 | 1.34-2.07 | 1.35-2.38 |  | 0.67-1.12 | 1.17-1.72 | 1.31-2.24 | 1.65-2.90 |
| Roll | 0.15-0.36 | 0.14-0.51 | 0.29-0.54 | 0.27-0.82 |  | 0.11-0.25 | 0.15-0.30 | 0.17-0.36 | 0.30-0.81 |
| Yaw | 0.03-0.12 | 0.08-0.17 | 0.09-0.18 | 0.07-0.16 |  | 0.05-0.19 | 0.07-0.17 | 0.07-0.21 | 0.05-0.16 |

Absolute values of deviations were used for statistical analysis. 95% CIs of the means were given for normally distributed data, while those of the medians were presented for the nonnormally distributed data (marked with ^#^). X, transversal translation of the dentition; Y, sagittal translation of the dentition; Z, vertical translation of the dentition.
